# Supplementary material for: A UMLS-based spell checker for natural language processing in vaccine safety
Source: BMC Med Inform Decis Mak. 2007 Feb 12;7:3. doi: 10.1186/1472-6947-7-3 (PMC1805499; doi:10.1186/1472-6947-7-3)
Supplement: Additional file 13 — RAP application directory. Sets up directory for RAP (RDF application for PHP) source code [file 1472-6947-7-3-S13.gz › rap/doc/phpdoc/elementindex_model.html]

xml version="1.0" encoding="iso-8859-1"?


## [model] element index

### Package indexes

- dataset
- infModel
- resModel
- ontModel
- syntax
- rdql
- utility

All elements
  

a
b
c
d
e
f
g
h
i
l
m
n
o
p
r
s
t
u
v
w

a

top

add
:   MemModel::add() in MemModel.php

    Adds a new triple to the MemModel without checking if the statement is already in the MemModel.

add
:   DbModel::add() in DbModel.php

    Add a new triple to this DbModel.

addModel
:   MemModel::addModel() in MemModel.php

    Adds another model to this MemModel.

addModel
:   DbModel::addModel() in DbModel.php

    Add the given model to this DbModel.

addNamespace
:   MemModel::addNamespace() in MemModel.php

    Adds a namespace and prefix to the model.

addNamespace
:   DbModel::addNamespace() in DbModel.php

    Adds a namespace and prefix to the model.

addParsedNamespaces
:   MemModel::addParsedNamespaces() in MemModel.php

    Adds the namespaces to the model. This method is called by the parser. !!!! addParsedNamespaces() not overwrites manual added namespaces in the model !!!!

addParsedNamespaces
:   DbModel::addParsedNamespaces() in DbModel.php

    Adds the namespaces to the model. This method is called by the parser. !!!! addParsedNamespaces() not overwrites manual added namespaces in the model !!!!

addWithoutDuplicates
:   MemModel::addWithoutDuplicates() in MemModel.php

    Checks if a new statement is already in the MemModel and adds the statement, if it is not in the MemModel.

addWithoutDuplicates
:   DbModel::addWithoutDuplicates() in DbModel.php

    Alias for the method add()

b

top

BlankNode
:   BlankNode::BlankNode() in Blanknode.php

    Constructor You can supply a label or You supply a model and a unique ID is gernerated

BlankNode
:   BlankNode in Blanknode.php

    An RDF blank node.

Blanknode.php
:   Blanknode.php in Blanknode.php

c

top

close
:   MemModel::close() in MemModel.php

    Close the MemModel and free up resources held.

close
:   DbModel::close() in DbModel.php

    Close this DbModel

close
:   DbStore::close() in DbStore.php

    Close the DbStore.

compare
:   Statement::compare() in Statement.php

    Compares two statements and returns integer less than, equal to, or greater than zero.

contains
:   MemModel::contains() in MemModel.php

    Tests if the MemModel contains the given triple.

contains
:   DbModel::contains() in DbModel.php

    Check if the DbModel contains the given statement

containsAll
:   MemModel::containsAll() in MemModel.php

    Determine if all of the statements in a model are also contained in this MemModel.

containsAll
:   DbModel::containsAll() in DbModel.php

    Determine if all of the statements in the given model are also contained in this DbModel.

containsAny
:   MemModel::containsAny() in MemModel.php

    Determine if any of the statements in a model are also contained in this MemModel.

containsAny
:   DbModel::containsAny() in DbModel.php

    Determine if any of the statements in the given model are also contained in this DbModel.

createTables
:   DbStore::createTables() in DbStore.php

    Create tables and indexes for the given database type.

d

top

datasetExists
:   DbStore::datasetExists() in DbStore.php

    Check if the Dataset with the given $datasetName is already stored in the database

DbModel
:   DbModel::DbModel() in DbModel.php

    Constructor Do not call this directly.

DbModel
:   DbModel in DbModel.php

    This class provides methods for manipulating DbModels from DbStore.

DbStore
:   DbStore::DbStore() in DbStore.php

    Constructor: Set the database connection with the given parameters.

DbStore
:   DbStore in DbStore.php

    DbStore is a persistent store of RDF data using relational database technology.

delete
:   DbModel::delete() in DbModel.php

    Remove this DbModel from database and clean up.

DbModel.php
:   DbModel.php in DbModel.php

DbStore.php
:   DbStore.php in DbStore.php

e

top

equals
:   Resource::equals() in Resource.php

    Checks if the resource equals another resource.

equals
:   Statement::equals() in Statement.php

    Checks if two statements are equal.

equals
:   MemModel::equals() in MemModel.php

    Checks if two models are equal.

equals
:   Literal::equals() in Literal.php

    Checks if ihe literal equals another literal.

equals
:   DbModel::equals() in DbModel.php

    Check if two models are equal.

equals
:   BlankNode::equals() in Blanknode.php

    Checks if two blank nodes are equal.

f

top

find
:   DbModel::find() in DbModel.php

    General method to search for triples in the DbModel.

find
:   MemModel::find() in MemModel.php

    General method to search for triples.

findAsIterator
:   MemModel::findAsIterator() in MemModel.php

    Returns a FindIterator for traversing the MemModel.

findCount
:   DbModel::findCount() in DbModel.php

    Search for triples and return the number of matches.

findCount
:   MemModel::findCount() in MemModel.php

    Searches for triples and returns the number of matches.

findFirstMatchingStatement
:   DbModel::findFirstMatchingStatement() in DbModel.php

    Search for triples and return the first matching statement.

findFirstMatchingStatement
:   MemModel::findFirstMatchingStatement() in MemModel.php

    Searches for triples and returns the first matching statement.

findForward
:   Model::findForward() in Model.php

    Searches for triples using find() and tracks forward blank nodes until the final objects in the retrieved subgraphs are all named resources.

findRegex
:   DbModel::findRegex() in DbModel.php

    Method to search for triples using Perl-style regular expressions.

findRegex
:   MemModel::findRegex() in MemModel.php

    Method to search for triples using Perl-style regular expressions.

findVocabulary
:   DbModel::findVocabulary() in DbModel.php

    Return all tripels of a certain vocabulary.

findVocabulary
:   MemModel::findVocabulary() in MemModel.php

    Returns all tripels of a certain vocabulary.

g

top

getBaseURI
:   Model::getBaseURI() in Model.php

    Return current baseURI.

getDatasetDb
:   DbStore::getDatasetDb() in DbStore.php

    Create a new instance of DatasetDb with the given $datasetName and load the corresponding values from the database.

getDatasetMem
:   ModelFactory::getDatasetMem() in ModelFactory.php

    Returns a NamedGraphSetMem.

getDatatype
:   Literal::getDatatype() in Literal.php

    Returns the datatype of the literal.

getDbModel
:   ModelFactory::getDbModel() in ModelFactory.php

    Returns a new DbModel using the database connection supplied by $dbStore.

getDbStore
:   ModelFactory::getDbStore() in ModelFactory.php

    Returns a database connection with the given parameters.

getDefaultDbModel
:   ModelFactory::getDefaultDbModel() in ModelFactory.php

    Returns a DbModel with the database connection defined in constants.php.

getDefaultModel
:   ModelFactory::getDefaultModel() in ModelFactory.php

    Returns a MemModel.

getID
:   BlankNode::getID() in Blanknode.php

    Returns the ID of the blank node.

getIndexType
:   MemModel::getIndexType() in MemModel.php

    Returns the indextype: -1 if there is no index, 0 if there are indices over S,P,O(separate),

    1. if there is an index over SPO, 2 if there is an index over SP and 3 if

    there is an index over SO.

getInfModelB
:   ModelFactory::getInfModelB() in ModelFactory.php

    Returns a InfModelB.

getInfModelF
:   ModelFactory::getInfModelF() in ModelFactory.php

    Returns a InfModelF.

getLabel
:   Literal::getLabel() in Literal.php

    Returns the string value of the literal.

getLabel
:   Resource::getLabel() in Resource.php

    Returns the label of the resource, which is the URI of the resource.

getLabel
:   BlankNode::getLabel() in Blanknode.php

    Returns the ID of the blank node.

getLabelObject
:   Statement::getLabelObject() in Statement.php

    Reurns the URI, text or bNode identifier of the statements's object.

getLabelPredicate
:   Statement::getLabelPredicate() in Statement.php

    Returns the URI of the statements's predicate.

getLabelSubject
:   Statement::getLabelSubject() in Statement.php

    Returns the URI or bNode identifier of the statements's subject.

getLanguage
:   Literal::getLanguage() in Literal.php

    Returns the language of the literal.

getLocalName
:   Resource::getLocalName() in Resource.php

    Returns the local name of the resource.

getMemModel
:   ModelFactory::getMemModel() in ModelFactory.php

    Returns a MemModel.

getMemModel
:   DbModel::getMemModel() in DbModel.php

    Create a MemModel containing all the triples of the current DbModel

getMemModelByRDQL
:   Model::getMemModelByRDQL() in Model.php

    Perform an RDQL query on this Model. Should work with all types of models.

getModel
:   DbStore::getModel() in DbStore.php

    Create a new instance of DbModel with the given $modelURI and load the corresponding values of modelID and baseURI from the database.

getNamedGraphDb
:   DbStore::getNamedGraphDb() in DbStore.php

    Create a new instance of namedGraphDb with the given $modelURI and graphName and load the corresponding values of modelID and baseURI from the database.

getNamespace
:   Resource::getNamespace() in Resource.php

    Returns the namespace of the resource. May return null.

getNewDatasetDb
:   DbStore::getNewDatasetDb() in DbStore.php

    Create a new instance of DatasetDb with the given $datasetName and insert the DatasetDb variables into the database.

getNewModel
:   DbStore::getNewModel() in DbStore.php

    Create a new instance of DbModel with the given $modelURI and insert the DbModel variables into the database.

getNewNamedGraphDb
:   DbStore::getNewNamedGraphDb() in DbStore.php

    Create a new instance of namedGraphDb with the given $modelURI and graphName and insert the DbModel variables into the database (not the graphName. This is only stored persistently, when added to dataset).

getObject
:   Statement::getObject() in Statement.php

    Returns the object of the triple.

getOntModel
:   ModelFactory::getOntModel() in ModelFactory.php

    Returns an OntModel.

getOntModel
:   Model::getOntModel() in Model.php

    Returns an OntModel with this model as baseModel.

getParsedNamespaces
:   DbModel::getParsedNamespaces() in DbModel.php

    Returns the models namespaces.

getParsedNamespaces
:   MemModel::getParsedNamespaces() in MemModel.php

    Returns the models namespaces.

getPredicate
:   Statement::getPredicate() in Statement.php

    Returns the predicate of the triple.

getResmodel
:   Model::getResmodel() in Model.php

    Returns a ResModel with this model as baseModel.

getResModel
:   ModelFactory::getResModel() in ModelFactory.php

    Returns a ResModel.

getStatementIterator
:   MemModel::getStatementIterator() in MemModel.php

    Returns a StatementIterator for traversing the MemModel.

getSubject
:   Statement::getSubject() in Statement.php

    Returns the subject of the triple.

getURI
:   Resource::getURI() in Resource.php

    Returns the URI of the resource.

h

top

hashCode
:   Statement::hashCode() in Statement.php

    Retruns the hash code of the triple.

i

top

index
:   MemModel::index() in MemModel.php

    Builds a search index for the statements in the MemModel.

intersect
:   DbModel::intersect() in DbModel.php

    Return a new MemModel containing all the statements which are in both this model and the given model.

intersect
:   MemModel::intersect() in MemModel.php

    Returns a new MemModel containing all the statements which are in both this MemModel and another.

isEmpty
:   DbModel::isEmpty() in DbModel.php

    Check if this DbModel is empty

isEmpty
:   MemModel::isEmpty() in MemModel.php

    Checks if MemModel is empty

isIndexed
:   MemModel::isIndexed() in MemModel.php

    Returns true if there is an index, false if not.

isSetup
:   DbStore::isSetup() in DbStore.php

    Checks if tables are setup for RAP

iterFind
:   MemModel::iterFind() in MemModel.php

    Returns a FindIterator for traversing the MemModel.

l

top

listModels
:   DbStore::listModels() in DbStore.php

    List all DbModels stored in the database.

Literal
:   Literal::Literal() in Literal.php

    Constructor

Literal
:   Literal in Literal.php

    An RDF literal.

load
:   Model::load() in Model.php

    Load a model from a file containing RDF, N3, N-Triples or a xhtml document containing RDF.

Literal.php
:   Literal.php in Literal.php

m

top

MemModel
:   MemModel in MemModel.php

    A MemModel is an RDF Model, which is stored in the main memory.

MemModel
:   MemModel::MemModel() in MemModel.php

    Constructor You can supply a base\_uri

Model
:   Model::Model() in Model.php

    Notice for people who are used to work with older versions of RAP.

Model
:   Model in Model.php

    Abstract superclass of MemModel and DbModel. A model is a programming interface to an RDF graph.

modelExists
:   DbStore::modelExists() in DbStore.php

    Check if the DbModel with the given modelURI is already stored in the database

ModelFactory
:   ModelFactory in ModelFactory.php

    ModelFactory is a static class which provides methods for creating different types of RAP models. RAP models have to be created trough a ModelFactory instead of creating them directly with the 'new' operator because of RAP's dynamic code inclusion mechanism.

MemModel.php
:   MemModel.php in MemModel.php

ModelFactory.php
:   ModelFactory.php in ModelFactory.php

Model.php
:   Model.php in Model.php

n

top

Node.php
:   Node.php in Node.php

Node
:   Node in Node.php

    An abstract RDF node.

o

top

object
:   Statement::object() in Statement.php

    Alias for getObject()

p

top

predicate
:   Statement::predicate() in Statement.php

    Alias for getPredicate()

putModel
:   DbStore::putModel() in DbStore.php

    Store a MemModel or another DbModel from a different DbStore in the database.

r

top

Resource.php
:   Resource.php in Resource.php

rdqlQuery
:   DbModel::rdqlQuery() in DbModel.php

    Perform an RDQL query on this DbModel.

rdqlQuery
:   MemModel::rdqlQuery() in MemModel.php

    Perform an RDQL query on this MemModel.

rdqlQueryAsIterator
:   MemModel::rdqlQueryAsIterator() in MemModel.php

    Perform an RDQL query on this MemModel.

rdqlQueryAsIterator
:   DbModel::rdqlQueryAsIterator() in DbModel.php

    Perform an RDQL query on this DBModel.

reify
:   MemModel::reify() in MemModel.php

    Reifies the MemModel.

reify
:   Statement::reify() in Statement.php

    Reifies a statement.

reify
:   DbModel::reify() in DbModel.php

    Reify the DbModel.

remove
:   MemModel::remove() in MemModel.php

    Removes the triple from the MemModel.

remove
:   DbModel::remove() in DbModel.php

    Remove the given triple from this DbModel.

removeNamedGraphDb
:   DbStore::removeNamedGraphDb() in DbStore.php

    Removes the graph with all statements from the database.

removeNamespace
:   MemModel::removeNamespace() in MemModel.php

    removes a single namespace from the model

removeNamespace
:   DbModel::removeNamespace() in DbModel.php

    removes a single namespace from the model

replace
:   DbModel::replace() in DbModel.php

    General method to replace nodes of a DbModel.

replace
:   MemModel::replace() in MemModel.php

    General method to replace nodes of a MemModel.

Resource
:   Resource in Resource.php

    An RDF resource.

Resource
:   Resource::Resource() in Resource.php

    Constructor Takes an URI or a namespace/localname combination

s

top

Statement.php
:   Statement.php in Statement.php

saveAs
:   DbModel::saveAs() in DbModel.php

    Saves the RDF,N3 or N-Triple serialization of the DbModel to a file.

saveAs
:   MemModel::saveAs() in MemModel.php

    Saves the RDF,N3 or N-Triple serialization of the MemModel to a file.

setBaseURI
:   MemModel::setBaseURI() in MemModel.php

    Set a base URI for the MemModel.

setBaseURI
:   DbModel::setBaseURI() in DbModel.php

    Set a base URI for the DbModel.

setDatatype
:   Literal::setDatatype() in Literal.php

    Sets the datatype of the literal.

setLanguage
:   Literal::setLanguage() in Literal.php

    Sets the language of the literal.

size
:   MemModel::size() in MemModel.php

    Number of triples in the MemModel

size
:   DbModel::size() in DbModel.php

    Return the number of statements in this DbModel

Statement
:   Statement::Statement() in Statement.php

    The parameters to constructor are instances of classes and not just strings

Statement
:   Statement in Statement.php

    An RDF statement.

subject
:   Statement::subject() in Statement.php

    Alias for getSubject()

subtract
:   DbModel::subtract() in DbModel.php

    Return a new MemModel that is the subtraction of another model from this DbModel.

subtract
:   MemModel::subtract() in MemModel.php

    Returns a new MemModel that is the subtraction of another model from this MemModel.

t

top

toString
:   MemModel::toString() in MemModel.php

    Short Dump of the MemModel.

toString
:   BlankNode::toString() in Blanknode.php

    Dumps bNode.

toString
:   Literal::toString() in Literal.php

    Dumps literal.

toString
:   Statement::toString() in Statement.php

    Dumps the triple.

toString
:   DbModel::toString() in DbModel.php

    Short dump of the DbModel.

toString
:   Resource::toString() in Resource.php

    Dumps resource.

toStringIncludingTriples
:   MemModel::toStringIncludingTriples() in MemModel.php

    Dumps of the MemModel including all triples.

toStringIncludingTriples
:   DbModel::toStringIncludingTriples() in DbModel.php

    Dump of the DbModel including all triples.

toStringObject
:   Statement::toStringObject() in Statement.php

    Reurns a toString() serialization of the statements's object.

toStringPredicate
:   Statement::toStringPredicate() in Statement.php

    Returns a toString() serialization of the statements's predicate.

toStringSubject
:   Statement::toStringSubject() in Statement.php

    Returns a toString() serialization of the statements's subject.

u

top

unite
:   MemModel::unite() in MemModel.php

    Returns a new MemModel that is the set-union of the MemModel with another model.

unite
:   DbModel::unite() in DbModel.php

    Return a new MemModel that is the set-union the model with another model.

v

top

visualize
:   Model::visualize() in Model.php

    Alias for RDFUtil::visualiseGraph(&$model, $format, $short\_prefix)

w

top

writeAsHtml
:   MemModel::writeAsHtml() in MemModel.php

    Writes the RDF serialization of the MemModel as HTML.

writeAsHtml
:   DbModel::writeAsHtml() in DbModel.php

    Write the RDF serialization of the \_DbModel as HTML.

writeAsHtmlTable
:   MemModel::writeAsHtmlTable() in MemModel.php

    Writes the RDF serialization of the MemModel as HTML table.

writeAsHtmlTable
:   DbModel::writeAsHtmlTable() in DbModel.php

    Write the RDF serialization of the DbModel as HTML table.

writeRdfToString
:   MemModel::writeRdfToString() in MemModel.php

    Writes the RDF serialization of the MemModel as HTML table.

writeRdfToString
:   DbModel::writeRdfToString() in DbModel.php

    Write the RDF serialization of the DbModel to string

a
b
c
d
e
f
g
h
i
l
m
n
o
p
r
s
t
u
v
w
